# An Outbreak of *tet*(X6)-Carrying Tigecycline-Resistant *Acinetobacter baumannii* Isolates with a New Capsular Type at a Hospital in Taiwan

**DOI:** 10.3390/antibiotics10101239

**Published:** 2021-10-12

**Authors:** Yu-Chia Hsieh, Jia-Wen Wu, Yi-Yin Chen, Tran Lam Tu Quyen, Wei-Chao Liao, Shiao-Wen Li, Yin-Cheng Chen, Yi-Jiun Pan

**Affiliations:** 1 Department of Pediatrics, Chang Gung Children’s Hospital, Chang Gung Memorial Hospital, Chang Gung University, College of Medicine, Taoyuan 333, Taiwan; yuchiahsieh@gmail.com (Y.-C.H.); d99445003@ntu.edu.tw (Y.-Y.C.); 2 Department of Microbiology and Immunology, School of Medicine, College of Medicine, China Medical University, Taichung 404, Taiwan; u105024609@cmu.edu.tw (J.-W.W.); tranlamtuquyen1991@gmail.com (T.L.T.Q.); 3 Molecular Medicine Research Center, Chang Gung University, Taoyuan 333, Taiwan; pettliao@mail.cgu.edu.tw (W.-C.L.); swli@cgu.edu.tw (S.-W.L.); cemily02@icloud.com (Y.-C.C.); 4 Linkou Medical Center, Department of Nephrology, Chang Gung Memorial Hospital, Taoyuan 333, Taiwan

**Keywords:** tigecycline, *Acinetobacter baumannii*, *tet*(X), *tet*(X6)

## Abstract

Dissemination of multidrug-resistant, particularly tigecycline-resistant, *Acinetobacter baumannii* is of critical importance, as tigecycline is considered a last-line antibiotic. Acquisition of *tet*(X), a tigecycline-inactivating enzyme mostly found in strains of animal origin, imparts tigecycline resistance to *A. baumannii*. Herein, we investigated the presence of *tet*(X) variants among 228 tigecycline-non-susceptible *A. baumannii* isolates from patients at a Taiwanese hospital via polymerase chain reaction using a newly designed universal primer pair. Seven strains (3%) carrying *tet*(X)-like genes were subjected to whole genome sequencing, revealing high DNA identity. Phylogenetic analysis based on the PFGE profile clustered the seven strains in a clade, which were thus considered outbreak strains. These strains, which were found to co-harbor the chromosome-encoded *tet*(X6) and the plasmid-encoded *bla*_OXA-72_ genes, showed a distinct genotype with an uncommon sequence type (Oxford ST793/Pasteur ST723) and a new capsular type (KL129). In conclusion, we identified an outbreak clone co-carrying *tet*(X6) and *bla*_OXA-72_ among a group of clinical *A. baumannii* isolates in Taiwan. To the best of our knowledge, this is the first description of *tet*(X6) in humans and the first report of a *tet*(X)-like gene in Taiwan. These findings identify the risk for the spread of *tet*(X6)-carrying tigecycline- and carbapenem-resistant *A. baumannii* in human healthcare settings.

## 1. Introduction

The emergence of multidrug-resistant Gram-negative bacteria poses a serious threat to global health. *Acinetobacter baumannii* is a troublesome nosocomial pathogen that causes pneumonia, sepsis, and wound and urinary tract infections, and particularly leads to severe disease and death in intensive care unit (ICU) patients [1,2,3,4,5,6]. Tolerance to desiccation and evasion of host immunity, together with the notorious antimicrobial resistance of *A. baumannii*, confer an advantage for the environmental and in-host survival of this microorganism. The spread of multidrug-resistant *A. baumannii* (MDRAB) has increased rapidly, and *A. baumannii* strains resistant to carbapenem, which has been used to treat MDRAB infections, has emerged [7,8,9,10,11,12,13]. Colistin and tigecycline are the two last-resort antibiotic options for treatment of infections caused by carbapenem-resistant *A. baumannii*. However, cases of colistin- or tigecycline-resistant *A. baumannii* infections have been reported worldwide [14,15,16,17].

Tigecycline is a tetracycline family antibiotic that inhibits bacterial protein synthesis by interacting with the 30S ribosomal subunit and inhibiting tRNA entry [18]. Compared to classical tetracyclines, tigecycline exhibits a higher affinity for ribosomes. However, tigecycline-resistant bacteria have emerged with the increasing use of tigecycline [19].

The primary mechanisms of tigecycline resistance are associated with mutations in the ribosome that block drug binding or result from overexpression of efflux proteins that actively pump out the drug. For example, mutations in *rpsJ*, which encodes ribosomal protein S10, alter the tigecycline-binding site and thus contribute to tigecycline resistance [20]. Another resistance mechanism involves the increased expression of efflux pumps, such as OqxAB, AcrAB-TolC, and AdeABC [21,22,23]. In addition, Tet proteins, including the tigecycline-modifying enzyme *tet*(X), the ribosomal protective protein *tet*(M), and the mutated *tet*(A) efflux pump, have also been reported to decrease tigecycline susceptibility [24,25]. 

*tet*(X), a flavin-dependent monooxygenase, catalyzes the cleavage of tigecycline via an oxygen-dependent mechanism. The *tet*(X) gene was first identified in Tn*4351* and Tn*4400* transposons in *Bacteroides fragilis* [26,27]. Subsequently, *tet*(X) and its variants have been reported in other *Bacteroides* species, *Enterobacteriaceae*, and *Acinetobacter* strains from animals and humans. Two of these variants, *tet*(X3) and *tet*(X6), have been found on both chromosomes and plasmids. Three variants, *tet*(X), *tet*(X1), and *tet*(X2), are chromosomally encoded, whereas *tet*(X4) and *tet*(X5) are found in plasmids [28,29,30,31,32,33,34,35,36,37,38,39]. Additional variants, *tet*(X7) to *tet*(X13), have been detected in environmental and human gut metagenomes [40]. Recently, a *tet*(X) variant, *tet*(X14), was reported in the chromosome of an *Empedobacter stercoris* isolate from a pig fecal sample [41]. Although *tet*(X)-bearing bacteria have been reported in China, Africa, America, and Europe [42,43,44,45], indicating the widespread dissemination of these genes, the number of cases is low, and most of them are isolated from animals.

Since *tet*(X)-like genes could spread between species through horizontal gene transfer, surveillance of the prevalence and abundance of these genes is important. To the best of our knowledge, *tet*(X) variants have not been documented in Taiwan. Thus, we aimed to investigate 228 tigecycline-non-susceptible *A. baumannii* clinical isolates collected in Taiwan for the presence of *tet*(X) variants.

## 2. Results

### 2.1. Screening of tet(X) Variants

We screened 228 non-repetitive clinical tigecycline-non-susceptible *A. baumannii* isolates in Taiwan for the presence of *tet*(X) variants via polymerase chain reaction (PCR) using a universal primer pair designed in this study (described in the Materials and Methods section). The PCR and sequencing results indicated the presence of *tet*(X)-like genes in seven strains, with a positive rate of 3%. The sources of the seven strains were blood (*n* = 2), sputum (*n* = 2), tissue (*n* = 1), urine (*n* = 1), and pleural effusion (*n* = 1) samples (Table S1).

### 2.2. Antimicrobial Susceptibility of Strains Carrying tet(X) Variants

The seven *tet*(X) variant-harboring strains were not susceptible to ceftazidime, ciprofloxacin, cefoperazone/sulbactam, cefepime, imipenem, meropenem, ampicillin–sulbactam, tigecycline, and tazobactam, but were susceptible to amikacin and colistin (data not shown). We further determined that the minimum inhibitory concentration (MIC) of tigecycline for the seven strains was 4–8 mg/L (regarded as tigecycline-resistant) (Table 1).

### 2.3. The Genomes of A. baumannii Isolates Carrying tet(X) Variants Are Highly Similar and Carry Two Plasmids

The genomes of the seven analyzed strains were almost identical (~100% identity and coverage) (BioProject ID: PRJNA672213; Accession Nos. CP064193–CP064204 and CP076736–CP076744), each comprising a circular chromosome and two plasmids of 112 kb and 9 kb. NCBI BLAST analysis showed that the chromosome shared high similarity with *A. baumannii* strain ab736 (Accession No. CP015121.1), which was isolated from a patient with bacteremia in the USA, and *A. baumannii* strain ZW85-1 (Accession No. CP006768) [46], which was isolated from the fecal sample of a patient with diarrhea in China (98.7% identity and 88% coverage for both isolates). Meanwhile, the best matches in the NCBI nucleotide database for the two plasmids were pCMCVTAb1-Ab59 [47] (Accession No. CP016299.1; 100% DNA identity and 98% coverage) and pAB-NCGM253 [48] (Accession No. AB823544; 100% DNA identity and coverage) for the 112 kb and 9 kb plasmids, which were obtained from clinical isolates in the USA and Japan, respectively.

### 2.4. Tigecycline-Resistant A. baumannii Isolates Carrying tet(X) Variants Also Carry Other Antimicrobial Resistance Genes

Antimicrobial resistance genes were identified using the Comprehensive Antibiotic Resistance Database (CARD). A total of 38 proteins exhibited >90% amino acid identity and coverage to proteins in the CARD database. We found a plasmid-encoded *bla*_OXA-72_ (*bla*_OXA-24_-like) carbapenemase gene (located in the 9 kb plasmid, which is almost identical to pAB-NCGM253, a common *bla*_OXA-72_-bearing plasmid found in several *Acinetobacter* spp. [49]) and a chromosome-encoded *bla*_OXA-66_ (*bla*_OXA-51_-like) carbapenemase gene (overexpression of *bla*_OXA-51_-like could confer carbapenem resistance), which may contribute to the carbapenem resistance of these isolates. The *adc-56*, a gene encoding extended-spectrum AmpC cephalosporinase, was found to confer cefepime resistance. In addition, genes encoding the aminoglycoside-modifying enzymes ANT(3′)-IIa, APH(3′)-Ib, APH(6)-Id, and APH(3′)-Ia; the chloramphenicol resistance gene *floR*; the sulfonamide resistance gene *sul2*; the tetracycline resistance genes *tet*(Y) and *tet*(X6); the *abaQ* gene encoding an efflux pump to mediate quinolone resistance; and the multidrug efflux pump-encoding genes such as *ade* were also identified.

### 2.5. The Tigecycline-Resistant A. baumannii Isolates Carry tet(X6) Genes

**Figure 1:**
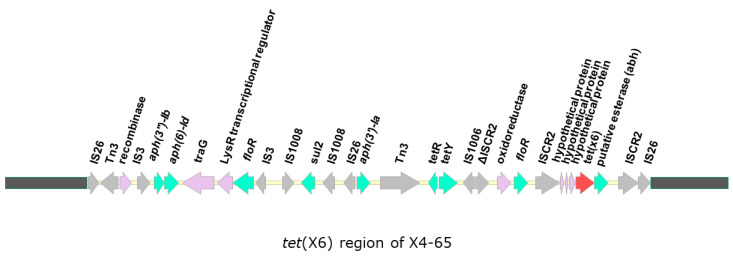
Genetic organization of the region surrounding *tet*(X6). A ~40 kb region containing *tet*(X6) unique to the strains analyzed in this study but absent in ab736 and ZW85-1 are shown. The open reading frames are indicated by arrows. The *tet*(X6) is colored in red, and other genes are colored according to their annotated functions: Green, antimicrobial resistance; grey, mobile element; purple, other functions.

In all seven sequenced strains, a *tet*(X6) gene was identified in the chromosome and was located in a ~40 kb region that is absent in *A. baumannii* ab736 and ZW85-1 from the NCBI database, which shared high genome identity with the strains analyzed in this study (Figure 1). It is noteworthy that this 40 kb region flanked by two directly repeated IS*26* sequences showed a higher G + C content (49.7%) than the rest of the chromosome (39%), indicating that this region may have originated from another source. 

**Figure 2:**
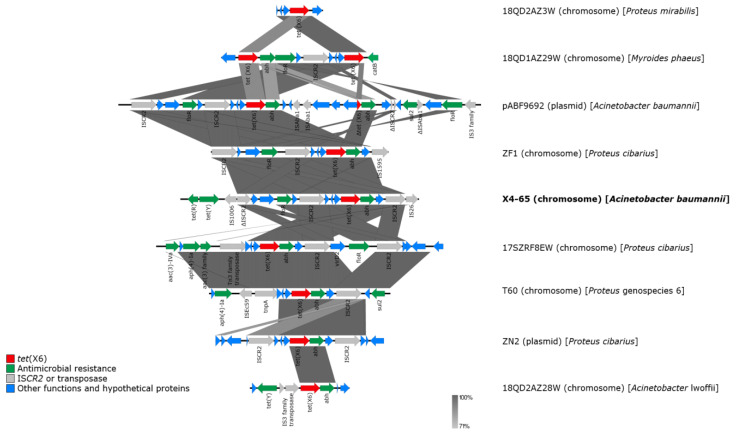
Comparison of *tet*(X6)-containing regions in different strains. Open reading frames are shown as arrows. Comparative analysis of DNA identity was performed using Easyfig 2.2.2. The *tet*(X6) genes are colored in red, and the other genes are colored according to their annotated functions: Green, antimicrobial resistance genes; grey, IS*CR2* or transposase-encoding genes; blue, other functions and hypothetical protein-encoding genes.

In addition to *tet*(X6), several other antibiotic resistance genes, including *aph(3′)-Ib*, *aph(6)-Id*, *aph(3′)-Ia*, *floR*, *sul2*, and *tet*(Y), were present in this region. Of note, several transposase-encoding genes were also identified, which suggests that transposition events occurred in this region and probably resulted in the accumulation of antimicrobial resistance genes. We further compared the genomic environments of previously reported *tet*(X6) genes in plasmids and chromosomes, including the sequences of plasmids from *A. baumannii* strain ABF9692 (plasmid pABF9692), *Proteus cibarius* strain ZN2, and the chromosomes of *Proteus* genospecies 6 strain T60, *P. cibarius* strain ZF1, *P. cibarius* strain 17SZRF8EW, *P. mirabilis* strain 18QD2AZ3W, *A. lwoffii* strain 18QD2AZ28W, *Myroides phaeus* strain 18QD1AZ29W, and *A. baumannii* strain X4-65 (this study) [35,38,50,51] (Figure 2). We found that, regardless of their location (plasmid or chromosome), *tet*(X6) were frequently associated with IS*CR2*, suggesting that IS*CR2* may contribute to the transmission of *tet*(X6). In addition, *tet*(X6) was found on an SXT/R391 integrative and conjugative element (ICE) in the chromosome of *Proteus* genospecies 6 (T60), an isolate from retail pork (the authors named the novel ICE ICEPgs6Chn1) [38]. However, the genetic environment of *tet*(X6) in our current study was different from that of ICEPgs6Chn1, and we did not find a complete ICE in the strains analyzed in this study, as the ICE finder tool (https://db-mml.sjtu.edu.cn/ICEfinder/ICEfinder.html (accessed on 5 October 2021)) and oriTfinder tool (https://tool-mml.sjtu.edu.cn/oriTfinder/oriTfinder.html (accessed on 5 October 2021)) could not detect an integrase gene, relaxase gene, *oriT*, or type IV secretion system-encoding genes.

### 2.6. The A. baumannii Isolates Showed No Evidence of Conjugal Transfer of tet(X6)

Although we did not identify a complete ICE associated with *tet*(X6), we examined whether *tet*(X6) could be transferred to *Escherichia coli* by conjugation. To ensure that the conjugation experimental procedures were successful, we used a *Klebsiella pneumoniae* strain that was able to transfer *the bla*_OXA-48_ gene to *E. coli* as a control. The results showed that the control *K. pneumoniae* can successfully transfer *the bla*_OXA-48_ gene to *E. coli*, and the transconjugants (*E. coli* J53-*bla*_OXA-48_) exhibited a higher imipenem MIC of ≥8 mg/L compared to the recipient (*E. coli* J53), with an MIC of 0.125 mg/L. However, no transconjugant was obtained for the *tet*(X6)-harboring *A. baumannii* strains under the conditions used in this study (Table 1 and Figure S1).

### 2.7. K Type and Sequence-Tyype (ST) of the Tigecycline-Resistant A. baumannii Isolates Carrying tet(X6)

**Figure 3:**
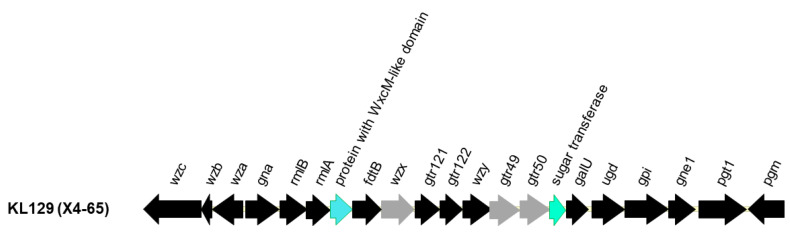
Capsular polysaccharide synthesis (*cps*) gene clusters in KL129. Capsular polysaccharide synthesis (*cps*) locus of KL129, which was identified in this study, was compared with that of BAL_329, an *A. baumannii* strain with KL60 capsular type (Accession No. MN148382.1). Open reading frames are shown as arrows. Comparing the two *cps* loci, conserved genes that shared > 95% amino acid identity with KL60 are shown in black. Genes that share 80–95% amino acid identity with KL60 are shown in grey, and genes sharing < 80% amino acid identity with KL60 are shown in green or blue for the corresponding genes.

The capsular types (K-types) of the seven strains were determined using Kaptive, a tool that predicts the K-type of *A. baumannii* strains based on the sequences of the capsular polysaccharide synthesis (*cps*) locus [52]. The results showed that these strains belong to a new K type, which we designated as KL129 (Accession No. MW353360), that is related to KL60. Two genes differed between KL60 and KL129: A sugar transferase gene and a gene encoding a WxcM-like domain-containing protein (Figure 3). The sugar transferase ItrA2 in KL60 and the corresponding protein in KL129 shared 77% amino acid identity at 95% coverage, whereas the WxcM-like domain-containing protein FdtE in KL60 and the corresponding protein in KL129 shared 73% amino acid identity at 99% coverage. In addition, sequence variations were found in other proteins: Wzx shared 89% amino acid identity at 99% coverage, Gtr49 shared 94% amino acid identity at 99% coverage, and Gtr50 shared 92% amino acid identity at 99% coverage. We also determined the STs of the strains analyzed in this study based on the obtained genome sequences. The results showed that they belonged to Oxford ST793/CC208 (previously denoted as CC92) and Pasteur ST723/CC2, a clonal complex (CC) that corresponds to international clone II (Figure S2).

### 2.8. Nosocomial Spread of Tigecycline-Resistant A. baumannii Isolates Carrying tet(X6)

**Figure 4:**
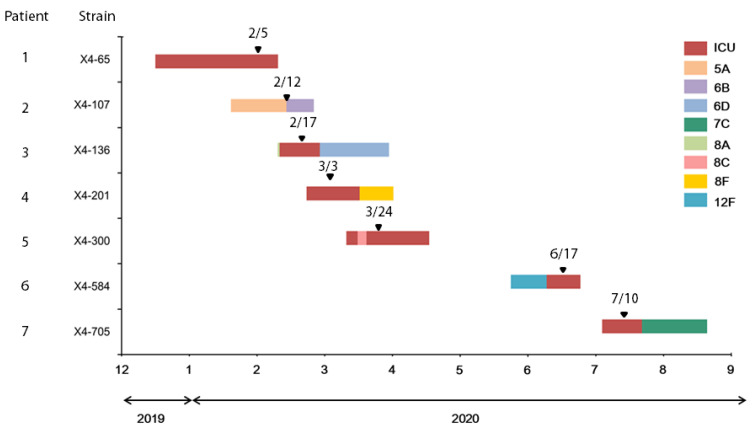
Medical history timeline of the patients with *tet*(X6)-carrying *A. baumannii* infection: Duration of hospital stay for each patient are shown. Intensive care unit is indicated in red, and general wards are shown in different colors representing different wards. The isolation time of *tet*(X6)-carrying *A. baumannii* was indicated by an arrowhead.

Six strains of *tet*(X6)-carrying *A. baumannii* were isolated from patients 1 and 3–7 in the same medical ICU; the last one (strain X4-107) was isolated from patient 2 in an orthopedic ward located on a separate floor of the same building (Figure 4 and Table S1). Patients 1, 3, and 5 had once been assigned to the same bed. The first strain (X4-65) was isolated (5 February 2020) from the sputum of patient 1 two months after admission to the ICU due to hepatic encephalopathy resulting from alcoholic liver cirrhosis. Patient 1 died of ventilator-associated pneumonia caused by *tet*(X6)-carrying *A. baumannii* three days later (8 February 2020). Strain X4-136 was isolated from patient 3, who was admitted on 10 February 2020, under the impression of pancreatitis with septic shock. The patient developed ventilator-associated pneumonia and central line-associated bloodstream infection caused by *tet*(X6)-carrying *A. baumannii* seven days later (17 February 2020). Approximately 52 days later, strain X4-300 was isolated from patient 5, who had hepatocellular carcinoma. The patient died of ventilator-associated pneumonia caused by *tet*(X6)-carrying *A. baumannii*. Strains from patients 4, 6, and 7, assigned to different beds in the same ICU, were isolated in March, June, and July, respectively. We presumed that this outbreak was caused by *tet*(X6)-carrying *A. baumannii* colonization in the environment of the medical ICU, where the healthcare worker(s) spread it to the orthopedic ward. We further performed in silico pulse field gel electrophoresis (PFGE) and constructed a phylogenetic tree based on the PFGE profile (Figure S4). The results showed that the seven strains collected in this study were clustered in a clade, indicating that these strains were clonally related.

## 3. Discussion

Since the first *tet*(X) was found in *B. fragilis* [26,27], several other *tet*(X) variants have been reported in different bacterial species, including *Acinetobacter* spp. [28,29,30,31,32,33,34,35,36,37,38,39,40,41]. As previous studies have indicated, *tet*(X)-carrying bacteria were detected more frequently in animal sources than in human sources. In 2019, one study detected *tet*(X3)/*tet*(X4) in 6.9% (73/1060) of animal samples compared to 0.07% (4/5485) of human samples [37]. Among these *tet*(X3)/*tet*(X4)-harboring strains, one was *A. baumannii*. A *tet*(X4)-harboring *A. baumannii* strain was identified among a group of 76 tigecycline-resistant *A. baumannii* (~1.3% positive rate of *tet*(X)-like genes) in an analysis of 1273 *A. baumannii* isolates from humans [37]. Another clinical survey reported in 2020 detected two *Acinetobacter* spp. with *tet*(X3) or *tet*(X5) in a group of 103 tigecycline-resistant strains among 2591 *Acinetobacter* spp. [53], with a similar positive rate of ~1.9% in tigecycline-resistant *Acinetobacter* spp. In the current study, we designed a universal primer pair to detect *tet*(X)-like genes, including *tet*(X)–*tet*(X14), in 228 non-repetitive clinical isolates of tigecycline-non-susceptible *A. baumannii*, and the results showed that 3% of the collected strains have *tet*(X)-like genes. Interestingly, all seven strains carried *tet*(X6) genes and were the same clone (Oxford ST793/CC208, Pasteur ST723/CC2). The *tet*(X6)-carrying *A. baumannii* strains were identified as a new capsular type (designated as KL129). These strains belong to a clonal complex linked with international clone II, which is a widely distributed clone [54]. However, the *tet*(X6)-carrying *A. baumannii* strains in this study represent distinct genotypes with an uncommon ST and a new K-type compared to previously reported common STs of MDRAB: Pasteur ST2, Oxford ST208, common K-types KL2 and KL22, and other documented ST/K types [52,55,56]. Herein, we demonstrated the nosocomial dissemination of this clone and suggested that the main source of transmission is the ICU environment and healthcare workers.

The co-existence of *tet*(X)-like genes and other antibiotic resistance genes has been reported in different bacterial strains isolated from animals or their environments, posing a serious threat to the clinical treatment of humans. A study of *E. coli* strains from an animal source possessing both *tet*(X4) and the colistin resistance gene *mcr-1* raised concerns, since tigecycline and colistin are regarded as last line drugs for the treatment of carbapenem-resistant bacteria [31]. A recent study documented an *A*. *baumannii* chicken isolate co-carrying a *tet*(X6) variant and the carbapenemase genes *bla*_NDM-1_ and *bla*_OXA-58_ [51]. Another study reported the co-occurrence of *tet*(X6) and the linezolid resistance gene *cfr* in *Proteus* spp. from swine farms [50]. In another study, *Acinetobacter* spp. harboring both *tet*(X) and *bla*_OXA-58_ were isolated from pigs [57]. In the current study, we found the co-carriage of carbapenemase gene *bla*_OXA-72_ and *tet*(X6) in *A. baumannii* strains isolated from patients. To the best of our knowledge, *tet*(X6) has been reported in the *Myroides*, *Proteus*, *E. coli*, *Providencia rettgeri*, and *A. baumannii* strains of animal origin [35,38,50,51,58], and this is the first description of *tet*(X6) in bacteria from human samples.

Although previous studies have reported that *tet*(X6) was associated with ICEs [38], we did not find a complete ICE in the region of the *tet*(X6) genes in the strains analyzed in this study. Concordantly, we failed to obtain *tet*(X)-containing transconjugants through conjugation, suggesting that other mechanisms, such as transformation, may be responsible for the transfer of *tet*(X)-containing DNA in the strains analyzed in this study. Of note, we found sequence similarity surrounding the genomic environments of reported *tet*(X6) genes in plasmids from *A. baumannii* and *Proteus cibarius*, and in the chromosomes of *P. mirabilis*, *P. cibarius Proteus* genospecies 6, *A. lwoffii*, *Myroides phaeus*, and *A. baumannii* (this study), suggesting that recombination events could occur between plasmids and chromosomes. Moreover, *tet*(X6) was associated with the presence of IS*CR2*, either at one or both ends, implying that IS*CR2* could play a role in the transmission of *tet*(X6).

Taken together, we demonstrated the presence of *tet*(X6) together with the carbapenemase gene *bla*_OXA-72_ in clinical isolates of *A. baumannii* and reported an outbreak at a hospital in Taiwan. The findings revealed evidence of clonal spread of *tet*(X6)-carrying tigecycline- and carbapenem-resistant *A. baumannii*.

Even though it seems that *tet*(X6) is restricted to certain clones and has not widely spread to large numbers of *A. baumannii* clinical isolates, it poses a real threat to healthcare systems. To control its dissemination, further investigations on its prevalence and distribution should be undertaken at different hospitals.

## 4. Materials and Methods

### 4.1. Tigecycline-Non-Susceptible A. baumannii Isolate Collection

A total of 228 non-redundant (when repetitive samples from the same patient were isolated, only the first sample was included) tigecycline-non-susceptible *A. baumannii* isolates were collected at Chang Gung Memorial Hospital, Lin Kou branch, a 3700-bed medical center in northern Taiwan, from January to September 2020. The MIC of tigecycline was determined using the broth dilution method according to Clinical and Laboratory Standards Institute (CLSI) recommendations. Since CLSI does not suggest breakpoints for tigecycline against *Acinetobacter* spp., the results were interpreted according to the European Committee on Antimicrobial Susceptibility Testing (EUCAST) v8.1 criteria for Enterobacterales (strains with an MIC ≤ 1 mg/L were defined as susceptible; MIC >2 mg/L were defined as resistant) [59].

### 4.2. PCR Detection of tet(X) Variants

To detect *tet*(X) variants in the isolates, we analyzed the strains for 19 *tet*(X) variant sequences (Table S2 and Figure S3). A pair of universal primers was designed to detect the 15 *tet*(X) variants, i.e., *tet*(X)–*tet*(X14) (Table S3). The PCR cycling program consisted of 96 °C for 3 min, followed by 30 cycles of 96 °C for 30 s, 52 °C for 30 s, and 72 °C for 30 s. Products with an expected size of ~800 bp were subjected to Sanger sequencing.

### 4.3. Bacterial Genome Sequencing and Analysis

Bacterial genomic DNA was extracted with a commercial kit (QIAamp DNA Mini Kit, Qiagen, Valencia, CA, USA) and subjected to nanopore sequencing (Good Future BioMed Inc., Kwenshan, Taoyuan, Taiwan). The sequencing library was prepared with a Rapid Barcoding Sequencing Kit (SQK-RKK004; Oxford Nanopore Technologies, Oxford, UK) according to the manufacturer’s instructions. Sequencing was performed on the GridION platform, and FlowCell (R9.4.1 FLO-MIN106D; Oxford Nanopore) was used to generate raw signal data. Base calling of the raw signal data was performed by Guppy (v3.2.1) in the HAC mode. The adaptors remaining in the base-called reads were trimmed with Porechop (v0.2.4). The clean reads were assembled into chromosome or plasmid contigs using Flye (v2.7). Any sequencing errors in the genome and plasmid contigs were first polished by four runs of Racon (v1.4.3). The remaining errors were removed by Medaka (v1.0) and validated by in-house scripts searching against the EMBL-EBI cDNA database. The protein-coding genes and rRNAs in the chromosomes and plasmids were annotated using the Prokka pipeline (v1.14.6). To identify antibiotic-resistance genes, the annotated genes were searched against the CARD using Diamond (v0.9.36).

### 4.4. Multilocus Sequence Typing (MLST) Analysis

Two schemes for ST assignment were used. The Pasteur scheme of MLST relies on seven housekeeping genes (*cpn60*, *gltA*, *recA*, *fusA*, *pyrG*, *rplB*, and *rpoB*) [60], and the Oxford scheme relies on *cpn60*, *gltA*, *recA*, *rpoD*, *gyrB*, *gdhB*, and *gpi* [61]. The target genes were extracted from the genome and subjected to ST analysis (www.pasteur.fr/mlst (accessed on 5 October 2021).). The global optimal eBURST algorithm was used to define the major clonal complexes of the strains.

### 4.5. Conjugation Assay

The conjugation assay was performed as described previously [62]. To ensure that the conjugation experimental procedures were successful, we used a donor, carbapenem-resistant *K. pneumoniae* strain (17CRE24), which was able to transfer the *bla*_OXA-48_ gene to *E. coli* by conjugation, collected from Tung’s Taichung Metro Harbor Hospital, Taiwan, as a control [63]. Seven tigecycline-resistant *A. baumannii* strains were used as donors, and sodium azide-resistant *E. coli* J53 was used as the recipient. The donors and recipients were cultured overnight at 37 °C in LB broth supplemented with 2 mg/L of tigecycline (for the seven tigecycline-resistant *A. baumannii* strains), 4 mg/L of imipenem (for the control *K. pneumoniae* strain), or 100 mg/L of sodium azide (for the *E. coli* J53 recipient). The donor and recipient cells were mixed at a ratio of 1:10 (100 μL donor and 1 mL recipient) and centrifuged at 8000× *g* for 5 min. The small pellet was resuspended in ~3 μL of LB broth, dropped onto a nitrocellulose membrane on an LB agar plate, and incubated overnight. The nitrocellulose membrane was transferred to a tube containing fresh LB broth and incubated at 37 °C for 30 min with shaking. Transconjugants were selected on LB agar containing 100 mg/L of sodium azide and supplemented with 2 mg/L of tigecycline or 2 mg/L of imipenem. Transconjugants were further plated on eosin methylene blue (EMB) agar to confirm *E. coli*, which produces a green metallic sheen on EMB. Furthermore, the successful transfer of genes was confirmed via PCR using specific primers (Table S3). The MICs of the successful transconjugants were determined.

### 4.6. In Silico Analysis of PFGE

The complete genomes were explored using in silico PFGE patterns via *AscI* restriction digestion [64]. Phylogenetic trees were generated to compare genetic relatedness and clonal assignment using the Dice distance from band pattern and agglomeration using the ward.D2 method [65,66].

## Figures and Tables

**Table 1:** MIC values of carbapenem-resistant *Klebsiella pneumoniae* 17CRE24, seven tigecycline-resistant *A. baumannii* strains, the recipient *E. coli* J53, and the transconjugants.

Strain Name	Bacteria Species	Description	Carbapenem/Tigecycline Resistance Genes	MIC (mg/L)	
				IMP	TIG
J53	*E. coli*	Recipient	-	0.125 (S)	0.125 (S)
17CRE24	*K. pneumoniae*	Donor	*bla*_OXA-48_	>16 (R)	ND
J53-*bla*_OXA-48_	*E. coli*	No. 1 transconjugant	*bla*_OXA-48_	8 (R)	ND
	*E. coli*	No. 2 transconjugant	*bla*_OXA-48_	8 (R)	ND
	*E. coli*	No. 3 transconjugant	*bla*_OXA-48_	8 (R)	ND
	*E. coli*	No. 4 transconjugant	*bla*_OXA-48_	8 (R)	ND
	*E. coli*	No. 5 transconjugant	*bla*_OXA-48_	8 (R)	ND
	*E. coli*	No. 6 transconjugant	*bla*_OXA-48_	8 (R)	ND
	*E. coli*	No. 7 transconjugant	*bla*_OXA-48_	>8 (R)	ND
	*E. coli*	No. 8 transconjugant	*bla*_OXA-48_	8 (R)	ND
	*E. coli*	No. 9 transconjugant	*bla*_OXA-48_	8 (R)	ND
	*E. coli*	No. 10 transconjugant	*bla*_OXA-48_	8 (R)	ND
	*E. coli*	No. 11 transconjugant	*bla*_OXA-48_	8 (R)	ND
	*E. coli*	No. 12 transconjugant	*bla*_OXA-48_	8 (R)	ND
X4-65	*A. baumannii*	Donor	*tet*(X6)	ND	8 (R)
X4-107	*A. baumannii*	Donor	*tet*(X6)	ND	8 (R)
X4-136	*A. baumannii*	Donor	*tet*(X6)	ND	8 (R)
X4-201	*A. baumannii*	Donor	*tet*(X6)	ND	8 (R)
X4-300	*A. baumannii*	Donor	*tet*(X6)	ND	8 (R)
X4-584	*A. baumannii*	Donor	*tet*(X6)	ND	8 (R)
X4-705	*A. baumannii*	Donor	*tet*(X6)	ND	4 (R)

Abbreviations: S, susceptible; R, resistant; ND, not determined; IMP, imipenem; TIG, tigecycline; MIC, minimum inhibitory concentration.

## Data Availability

The data used to support the findings of this study are available from the corresponding author upon request.

## Supplementary Materials

The following are available online at https://www.mdpi.com/article/10.3390/antibiotics10101239/s1, Table S1: the seven *tet*(X)-harboring strains and patients; Table S2: *tet*(X) variants included for sequence analysis, Table S3: Primer pairs used for PCR amplification, Figure S1: PCR confirmation of transconjugants, Figure S2: Clonal complexes of tigecycline-resistant *A. baumannii* isolates carrying *tet*(X6), Figure S3: Alignment of *tet*(X) variants and primer design, Figure S4: Phylogenetic tree of *A. baumannii* isolates based on PFGE profile.
