# Supplementary material for: An Outbreak of tet(X6)-Carrying Tigecycline-Resistant Acinetobacter baumannii Isolates with a New Capsular Type at a Hospital in Taiwan
Source: Antibiotics (Basel). 2021 Oct 12;10(10):1239. doi: 10.3390/antibiotics10101239 (PMC8532604; doi:10.3390/antibiotics10101239)
Supplement: Supplementary file 1 [file antibiotics-10-01239-s001.zip › antibiotics-1398827-supplementary.pdf]

Table S1. Information on the seven *tet*(X)-harboring strains isolated from patients

Patient number	Patient age (y/o)	Gender	Name of strain	Isolation Source	Isolation date
1[a]	51	Male	X4-65	SP	Feb-05
2	26	Male	X4-107	TS	Feb-12
3[a]	43	Female	X4-136	B	Feb-17
4	63	Female	X4-201	B	Mar-03
5[a]	75	Male	X4-300	SP	Mar-24
6	80	Female	X4-584	U	Jun-17
7	50	Female	X4-705	PL	Jul-10

[a], the same bed; SP, sputum; TS, Tissue; B, Blood; U, Urine; PL, Pleural effusion

Table S2. The *tet*(X) variants included in the sequence analysis

***tet*(X) variant**	**Accession number**	**Reference**
tet(X)	M37699	[33]
tet(X1)	AJ311171	[29]
tet(X2)	AJ311171	[29]
tet(X3)	MK134375	[37]
*tet(*X3.2)	CP043635	[36]
*tet(*X4)	MK134376	[37]
tet(X4)	NG_065852	[37]
*tet(*X5)	CP040912	[30]
*tet(*X6)	MN507533	[38]
*tet(*X7)	KU547176.1	[40]
*tet(*X8)	KU548516.1	[40]
*tet(*X9)	MT292309	[40]
*tet(*X10)	KF628469.1	[40]
*tet(*X11)	MT292307	[40]
*tet(*X12)	KU548377.1	[40]
*tet(*X13)	KU547125.1	[40]
*tet(*X14)	CP004020	[28]
*tet(*X14)	CP006649	[41]
*tet(*X14.2)	CP003787	[41]

Table S3. Primer pairs used for PCR amplification

Primer	Sequence (5′-3′)	Fragment	Purpose and reference
tet(X)-uni F	TTCAGGTCAGGAAGCAATGAAAA	780 bp	*tet*(X) variants detection [this study]
tet(X)-uni R	TTTACGCCTTGTCCTGCAAAAGG		
OXA-48-F	GCGTGGTTAAGGATGAACAC	438 bp	$\textit{bla}_{\text{OXA-48}}$ detection [60]
OXA-48-R	CATCAAGTTCAACCCAACCG		

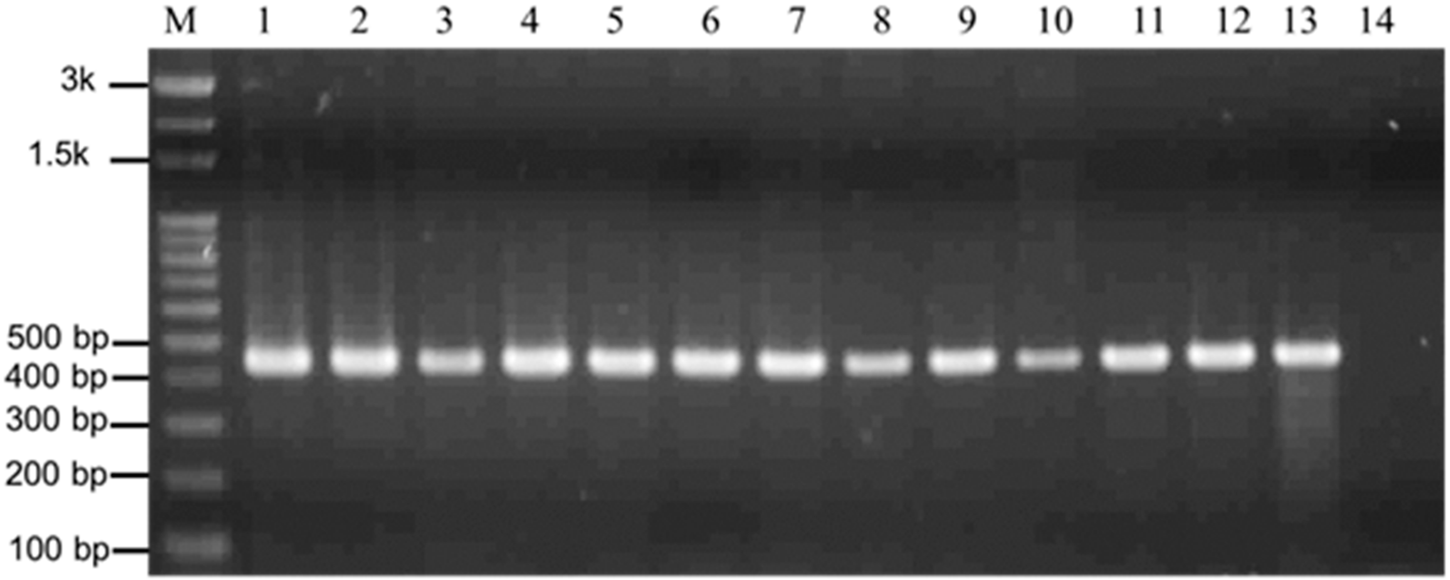

Figure S1. PCR confirmation of transconjugants

Seven tigecycline–non-susceptible *Acinetobacter baumannii* strains and one carbapenem-resistant *Klebsiella pneumoniae* strain (17CRE24, a positive control) were used as donors, and sodium azide–resistant *Escherichia coli* J53 was used as the recipient. Transconjugants were selected on LB agar containing 100 mg/L sodium azide supplemented with 2 mg/L tigecycline or 2 mg/L imipenem (for control). For the control experiment, transconjugants were grown on selective agar, and 12 colonies were selected for the detection of *the bla*$_{OXA\text{-}48}$ gene using PCR. In contrast, no transconjugant was obtained for the conjugation experiment of the *tet*(X6)-harboring *A. baumannii* strains and J53. Lanes 1–12, transconjugants; Lane 13, 17CRE24 (donor, a positive control). Lane 14, *E. coli* J53 (recipient, negative control). Product size is 438 bp.

Oxford ST analysis

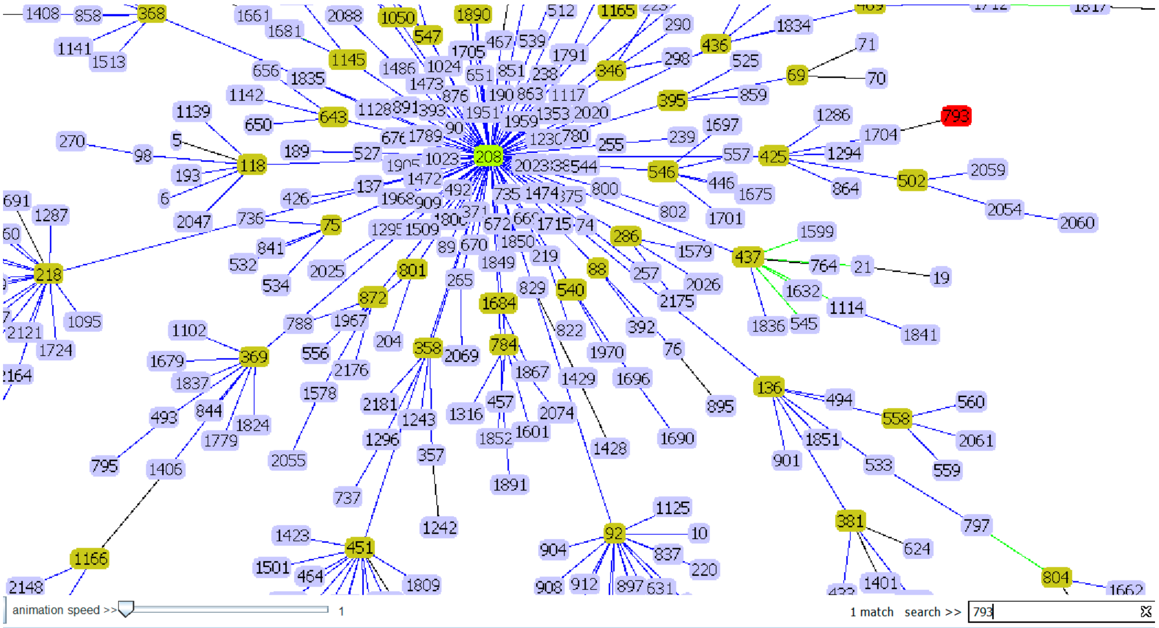

Pasteur ST analysis

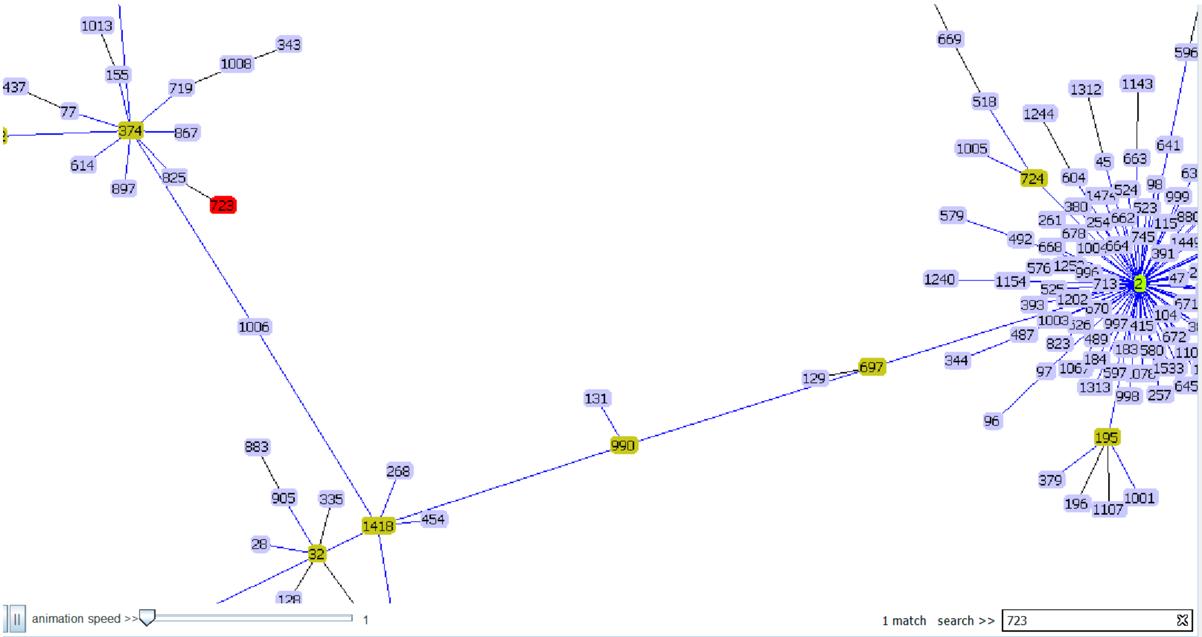

Figure S2. Clonal complexes of tigecycline-resistant *A. baumannii* isolates carrying *tet*(X6)

Global optimal eBURST analysis was performed with either Oxford or Pasteur sequence type (ST) database to verify the clonal complexes to which the isolates identified in the present study belong to. The sequence type was linked to another if they differed in only one of the seven multilocus sequence typing loci, which are called single-locus variants. The central ST type in the cluster was defined as the founder of the clonal complex. The results showed that Oxford ST793 belongs to CC208 (previously denoted as CC92) and Pasteur ST723 belongs to CC2.

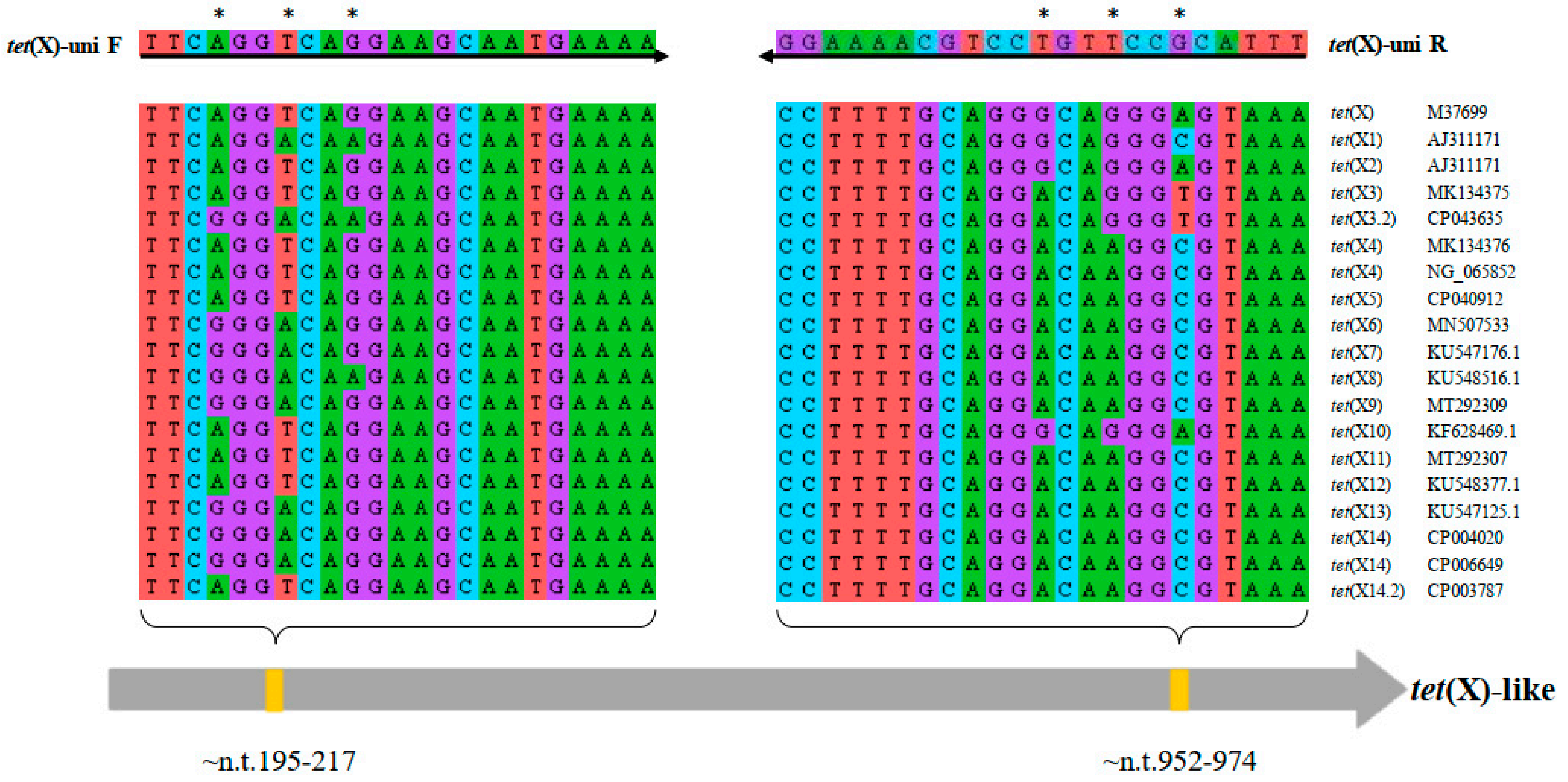

Figure S3: Alignment of *tet*(X) variants and primer design

A total of 19 *tet*(X) variant sequences were analyzed, and two primers were designed for the detection of *tet*(X)-like genes. Primers were perfectly matched to *tet*(X) variants; however, *tet*(X)-uni F has two mismatches with *tet*(X1), *tet(*X6), *tet*(X7), *tet*(X9), *tet*(X13), and *tet*(X14), and three mismatches with *tet*(X3.2). Meanwhile, *tet*(X)-uni R has two mismatches with *tet*(X1), *tet*(X3), and *tet* (X3.2); and three mismatches with *tet*(X), *tet* (X2), and *tet*(X10). * positions possibly with mismatches

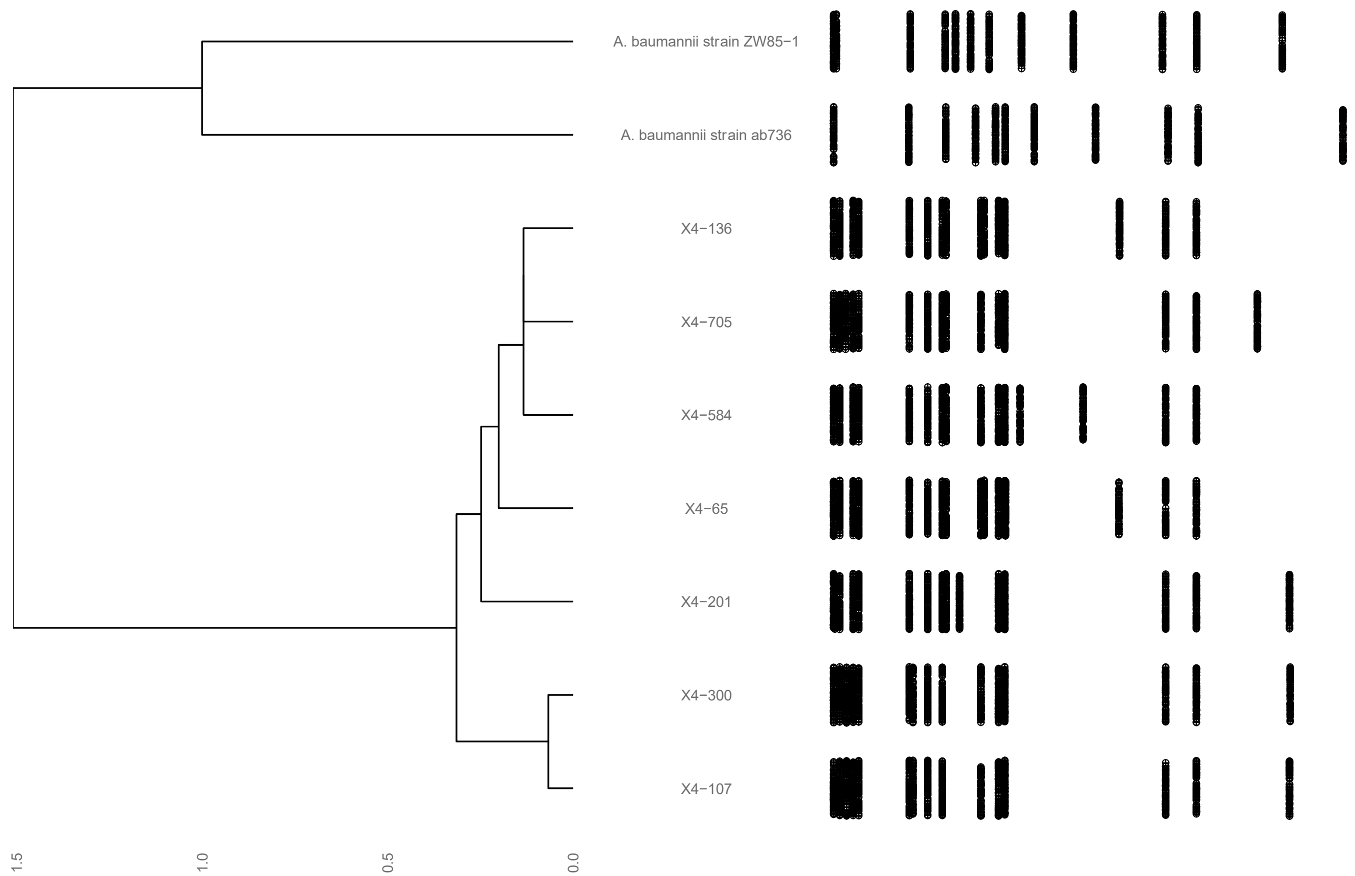

Figure S4. Phylogenetic tree of *A. baumannii* isolates based on pulsed-field gel electrophoresis (PFGE) profile

In silico digestion and PFGE were performed using *AscI* restriction enzyme. Phylogenetic trees were constructed using the Dice distance from the band pattern and agglomeration using the ward.D2 method. *A. baumannii* ab736 and ZW85-1 from the NCBI database were also included for comparison.
